# 

**Published:** 1871-07

**Authors:** 


### A WORD TO OUR SUBSCRIBERS.

Owing to unavoidable circumstances, our JOURNAL has been delayed in its appearance at the usual time. We take pleasure in saying, however, that our publisher will use his best exertions to have the JOURNAL issued promptly. Should any of our subscribers fail to get the COMPANION regularly they will confer a favor by informing us of the fact.

---

WITH all men who believe it honorable to slander private and professional character, Bodies or Associations, will regard it as dishonorable to introduce facts drawn from conversations to disprove such slanders; but it will never be the verdict of honorable men.

### BOOKS AND PAMPHLETS.

A Practical Treatise on the Diseases of Infancy and Childhood: By Thomas Hawkes Tanner, M. D., F. L. S. Author of the Practice of Medicine, Index of Diseases, and their treatment, etc. Third American Edition, from the last London edition, and enlarged, by Alfred Meadows, M. D., Lond. Member of the Royal College of Physicians, &c. Philadelphia: Lindsay & Blakison, 1871.

We have received from the Publishers, through Messrs. Lynch & Co., Atlanta, Ga., the above volume—price $3,50. The value of the work before us cannot be too highly estimated, when it is remembered that the diseases of children constitute a large element in the practice of every physician, requiring careful study and the most industrious research in order to successfulty meet them by appropriate treatment. The practitioner will be furnished very great aid by availing himself of this excellent treatise—perhaps the very best (as well as latest), on the subject.

The Causation, Course and Treatment of Reflex Insanity in Women, by Horatio Robinson Storer, M. D. LL. B., of Boston, Surgeon to St. Elizabeth's and St. Francis Hospitals for Women, Professor of Obstetrics and the Diseases of Women in Berkshire Medical College, etc., etc., etc. Boston: Lee & Shepard, Publishers, 1871. We have received from the author the above-mentioned work. As a Gynæcologist, Dr. Storer occupies a proud and distinguished position, with the first of his profession in the land. In bringing before the profession the importance and frequency of "Reflex Insanity in Women," he may very justly be considered the pioneer. That he has successfully established the truth of his position, no fair mind will question. His little volume is highly instructive, extremely "readable" and abounds in valuable thoughts and hints to the practical physician and gynæcologist. We hope the promise made by the author will be soon verified—i. e. that he furnish "the profession with a work covering the entire ground so much demanded by the importance of the subject of the reflex Insanity of Women." In the meantime, we trust the profession will do themselves the pleasure to read the above volume—being the best one extant upon the subject.

Diseases of the Womb, Uterine Catarrh, frequently the cause of sterility. New treatment by H. E. Gantillon, M. D., is the title of a pamphlet of 54 pages, and published by James Campbell, 18 Tremont Street, Boston, 1871. Price 50 cents. This is a very interesting pamphlet, and the author hopes it will be received by his medical brethren as an encouragement to the efforts he is making to bring forward a much more important work on the same subject. His treatment consists in intra-uterine injections by an instrument of his own invention, the mechanism of which he asserts, allows the injected liquid to retrograde through the orifice of the os uteri without any difficulty. He thinks it entirely safe to inject fluids into the cavity of the womb with this instrument, and gives a number of cases safely and rapidly cured by its use.

The Treatment of Paralysis by Hypodermic Injections of Strychnia. By Reuben A. Vance, M. D. D. Appleton & Co., 90, 92 and 94, Grand Street, New York. This is a pamphlet of very great practical importance, and reveals, most satisfactorily, the value of the use of strychnia hypodermically for paralysis, over its administration by the mouth.

The Modern Operation for Cateract: A lecture delivered at the Harvard Medical School, April 5, 1871, with an analysis of sixty-one operations. By Hasket Derby, M. D., University Lectures on Opthalmology, and Surgeon to the Massachusetts Charitable Eye and Ear Infirmary, Boston, 1871. This is a very useful pamphlet, and one that the surgeon will highly appreciate. We would like to give a more extended notice of this pamphlet, but our want of space forbids.

The Detection of Criminal Abortion, by Ely Van de Warker, M. D., Syracuse, N. Y. Boston: James Campbell, Publisher,

1871. We acknowledge the reception of the above pamphlet, with the compliments of the author. We have read this pamphlet with great interest. We do not think, however, that criminal abortion prevails to any considerable extent in the South—at least in this portion of it. The pamphlet will be found very interesting and instructive.

On the management of Lumber and Psoas Abscess. By Chas. F. Taylor, M. D. This will be found a very satisfactory and instructive monograph upon the subject of which it treats.

Hygiene in its relations to Therapeutics. A paper read before the N. Y. Medical Association. By Alfred L. Carroll, M. D. A very important subject, and one ably discussed by the author.

